# Affective and cognitive rather than somatic symptoms of depression predict 3-year mortality in patients on chronic hemodialysis

**DOI:** 10.1038/s41598-018-24267-5

**Published:** 2018-04-12

**Authors:** Hui-Teng Cheng, Miao-Chun Ho, Kuan-Yu Hung

**Affiliations:** 1 Division of Nephrology, Department of Internal Medicine, National Taiwan University Hospital, Hsin-Chu Branch, Hsin Chu City, Taiwan; 2 Department of Nursing, National Taiwan University Hospital, Hsin-Chu Branch, Hsin Chu City, Taiwan

## Abstract

Depression is more common in many medical conditions than among the general population and is associated with an increased risk of mortality. We aimed to determine whether somatic symptoms of depression were more predictive of mortality than affective and cognitive symptoms in hemodialysis patients. We conducted a prospective cohort study in which the survival outcomes of 151 subjects were followed for more than 3 years. Depression was assessed with the Taiwanese Depression Questionnaire (TDQ). Subjects with TDQ scores 19–54 (correlated with clinically significant depressive symptoms) and those with scores 15–18 had higher 3-year mortality rates than the two groups with lower scores (40.0%, 46.7%, 16.0% and 19.6%, p = 0.021, ANOVA). Affective and cognitive symptoms, including sadness, tenseness, indecisiveness and low self-confidence, and one somatic item (bodily discomfort) were associated with mortality. Affective and cognitive symptoms affected quality of life more than somatic symptoms. The somatic subscale was associated with female gender, low income and education, dialysis vintage, and low serum creatinine and albumin levels, whereas the affective and cognitive subscale was associated with less education and a low serum albumin level. In conclusion, affective and cognitive symptoms of depression may better predict long-term mortality in patients undergoing chronic hemodialysis than somatic symptoms.

## Introduction

Depression is a mood disorder with a prevalence in the general population ranging from 1.6% in China^1^ to 5.0% in the United Kingdom^2^ and 9.0% in the United States^3^. Depression is even more common in patients with certain organic illnesses, such as congestive heart failure (63%)^4^, malignancy (20.7%)^5^ and diabetes (20–25%)^6^. Coexistence of the diseases with depression is associated with many adverse outcomes, including increased mortality^4,6,7^. Depression is also very common in patients with end-stage renal disease who are undergoing hemodialysis, with prevalence rates ranging from 5% to 64.5%^8^. Numerous studies have shown an association between depression and higher mortality rates in hemodialysis patients^9–12^, with few exceptions^13,14^. Depression is also associated with a poor quality of life^15–17^.

The symptomatology of depression includes somatic complaints and non-somatic disturbance; the latter includes affective and cognitive impairments, such as sadness, negative thoughts, anhedonia or loss of interest, indecisiveness and suicidal ideation^18^. Somatic symptoms of depression include fatigue, poor appetite, concentration difficulty, bodily discomfort, poor memory or forgetfulness and loss of sexual drive^19^. The somatic symptoms of depression overlap with a variety of other disorders that may be present in patients on dialysis, such as hyperuremia^20^ and sleep disorders^21,22^. In order to make a definite diagnosis of depression, low mood or decreased interest must be present. Otherwise, somatic symptoms such as fatigue, poor appetite and forgetfulness may be caused by inadequate dialysis or dementia^23^. The severity of somatic symptoms has been shown to be a better predictor of mortality than affective and cognitive symptoms in patients with myocardial infarction. In these patients, it is thought that somatic symptoms directly reflect physiological conditions^24^. However, the comparison has not been performed for other types of diseases.

For hemodialysis patients, Kimmel *et al*. demonstrated an association between mortality and the Cognitive Depression Index (CDI), which is a 15-question subscale of the 21-question Beck Depression Inventory (BDI) that excludes items related to somatic symptoms^25^. In another smaller series that included patients undergoing hemodialysis and peritoneal dialysis, Peterson *et al*. showed an association between higher CDI scores and lower survival rates^26^. Kellerman *et al*. also demonstrated this association in patients with chronic kidney disease before entering dialysis^27^. To the best of our knowledge, there is no literature examining the association between somatic symptoms of depression and mortality in patients on chronic hemodialysis. In addition, if such an association exists, whether somatic symptoms are a better predictor of mortality than the affective and cognitive symptoms remains to be determined.

To answer these two questions, we conducted a prospective cohort study to determine how depressive symptoms affected 3-year mortality and quality of life, as the primary and secondary outcomes, respectively. We used the Taiwan Depression Questionnaire to measure the degree of various somatic, affective and cognitive symptoms. The questionnaire is similar to the commonly used Beck Depression Inventory, but has been adapted to be culturally relevant^28^. The classification between somatic and affective/cognitive items is largely based on the Cognitive Depression Index, a 15-item subscale that is included within the 21-item Beck Depression Inventory^25^. We also investigated the associations between the depressive symptoms and various demographic and biochemical parameters.

## Results

### Depressive symptoms are associated with higher mortality rates, lower quality of life, and low serum creatinine and serum albumin levels in patients on dialysis

In the current cohort, 43% of the subjects had scores higher than 19, regarded as having depression^28^. These subjects had higher 3-year mortality rates than the patients with normal scores (40.0% versus 23.3%, Table 1). Remarkably, a higher mortality rate was found in subjects with the Taiwanese Depression Questionnaire (TDQ) scores higher than 15 (Table 2). Patients with higher TDQ scores also reported a significantly poorer quality of life (Tables 1 and 2). The subjects with the highest TDQ scores (>19) had lower serum creatinine and albumin levels than those with normal TDQ scores (0–8) (Table 2).

**Table 1:** Demographic and biochemistry characteristics of depressed and non-depressed subjects based on the Taiwanese Depression Questionnaire (TDQ) score (n = 151).

	Total (n = 151)	No depression (TDQ 0–18) (n = 86, 57.0%)	Depression (TDQ 19–54) (n = 65, 43.0%)	*p* value of *t*-test, z-test or Fisher’s exact test
3-year mortality	46 (30.5%)	20 (23.3%)	26 (40.0%)	**0.027**
Age	64.6 ± 13.9	63.5 ± 14.2	66.1 ± 13.5	0.984
Gender (woman %)	77 (50.1%)	39 (45.3%)	38 (58.5%)	0.109
Quality of life score	79.87 ± 16.29	89.6 ± 12.83	66.9 ± 10.21	**0.058**
% QOL score ≤ 65	30 (19.9%)	2 (2.3%)	28 (43.1%)	**0.000**
Marital status				0.752
married	118 (78.1%)	68 (79.1%)	50 (76.9%)	
other	33 (21.9%)	18 (20.9%)	15 (23.1%)	
Education				0.073
≤6 years	85 (56.3%)	43 (50.0%)	42 (64.6%)	
>6 years	66 (43.7%)	43 (50.0%)	23 (35.4%)	
Occupation				0.115
unemployed	132 (87.4%)	72 (83.7%)	60 (92.3%)	
employed	19 (12.6%)	14 (16.3%)	5 (7.7%)	
Religion				0.558
nil	49 (32.5%)	26 (30.2%)	23 (35.4%)	
Buddhism	65 (43.0%)	39 (45.3%)	26 (40.0%)	
Christianity	3 (2.0%)	2 (2.3%)	1 (1.5%)	
Catholicism	12 (7.9%)	5 (5.8%)	7 (10.8%)	
Taoism	21 (13.9%)	14 (16.3%)	7 (10.8%)	
miscellaneous	1 (0.7%)	0 (0.0%)	1 (1.5%)	
Monthly income (USD)				0.256
<300	87 (57.6%)	46 (53.5%)	41 (63.1%)	
300–1000	37 (24.5%)	20 (23.3%)	17 (26.2%)	
1000–2000	17 (11.3%)	13 (15.1%)	4 (6.2%)	
>2000	10 (6.6%)	7 (8.1%)	3 (4.6%)	
Living				0.990
alone	5 (3.3%)	3 (3.5%)	2 (3.1%)	
with family	139 (92.1%)	79 (91.9%)	60 (92.3%)	
other	7 (4.6%)	4 (4.7%)	3 (4.6%)	
Diabetes mellitus	84 (55.6%)	49 (57.0%)	35 (53.8%)	0.704
BUN (mg/dL)	75.2 ± 20.9	78.7 ± 20.4	70.9 ± 21.1	0.692
Creatinine (mg/dL)	10.34 ± 2.58	10.90 ± 2.5	9.70 ± 2.50	0.968
Sodium (mmol/L)	136.9 ± 4.1	137.2 ± 3.5	136.5 ± 4.8	**0.015**
Potassium (mmol/L)	4.44 ± 0.97	4.51 ± 0.84	4.35 ± 1.11	0.641
Calcium (mg/dL)	9.30 ± 0.87	9.26 ± 0.87	9.36 ± 0.87	0.561
Phosphate (mg/dL)	4.90 ± 1.47	5.18 ± 1.46	4.52 ± 1.40	0.747
Ca x P product	45.70 ± 14.65	48.00 ± 14.30	42.66 ± 14.66	0.573
Albumin (g/dL)	3.65 ± 0.37	3.73 ± 0.36	3.53 ± 0.35	0.772
Glucose (mg/dL)	153.2 ± 65.8	152.2 ± 59.7	154.5 ± 73.5	0.458
GOT (IU/L)	18.4 ± 10.1	18.7 ± 11.2	18.2 ± 8.5	0.676
GPT (IU/L)	19.1 ± 20.0	19.4 ± 23.1	18.8 ± 15.1	0.769
Kt/V of urea	1.34 ± 0.22	1.37 ± 0.23	1.30 ± 0.20	0.921
Dialysis vintage (years)	4.5 ± 3.9	4.5 ± 3.8	4.7 ± 4.1	0.556
ALKP (U/L)	307.6 ± 138.6	309.2 ± 146.5	305.6 ± 128.4	0.704
Triglyceride (mg/dL)	169.8 ± 111.9	164.8 ± 98.5	176.3 ± 128.1	0.348
Cholesterol (mg/dL)	159.8 ± 34.9	159.9 ± 36.3	159.7 ± 33.2	0.461
Ferritin (ng/mL)	460.8 ± 444.5	475.4 ± 554.2	441.4 ± 233.5	0.341
i-parathyroid hormone (pg/mL)	338.0 ± 271.4	361.7 ± 289.5	306.7 ± 244.0	0.512
Hemoglobin (g/dL)	10.78 ± 1.51	10.78 ± 1.53	10.78 ± 1.50	0.844
Platelet (k/µL)	177.1 ± 64.9	176.9 ± 59.8	177.3 ± 71.6	0.676

Data are presented as the mean ± standard deviation, or the count number (percentage). The p values are from the analyses between the participants with and without depression. ALKP: alkaline phosphatase, BUN: blood urea nitrogen, Ca x P: calcium and phosphate product, GOT: glutamic oxaloacetic transaminase, GPT: glutamic pyruvic transaminase, i-parathyroid hormone: intact parathyroid hormone, Kt/V: dialysis adequacy by dialyzer clearance of urea (K) times time (t) divided by volume (V) of the urea distribution, USD: US dollars.

**Table 2:** Demographic characteristics based on the Taiwanese Depression Questionnaire (TDQ) score (n = 151).

Variables	Total (n = 151)	(1) TDQ 0–8 (n = 46, 30.5%)	(2) TDQ 9–14 (n = 25, 16.6%)	(3) TDQ 15–18 (n = 15, 9.9%)	(4) TDQ 19–54 (n = 65, 43.0%)	*p* value
3-year mortality	46 (30.5%)	9 (19.6%)	4 (16.0%)	7 (46.7%)	26 (40.0%)	**0.021** **(1)** < **(3)** **(2)** < **(3)** **(1)** < **(4)** **(2)** < **(4)**
Age	64.6 ± 13.9	60.3 ± 14.4	63.2 ± 13.5	73.9 ± 9.5	66.1 ± 13.5	**0.007** **(1)** < **(3)**
Gender (women %)	77 (50.1%)	18 (39.1%)	13 (52.0%)	8 (53.3%)	38 (58.5%)	0.253
Quality of life score	79.87 ± 16.29	95.2 ± 12.3	85.7 ± 9.9	79.3 ± 9.8	66.9 ± 10.2	**0.000** **(1)** > **(2)** = **(3)** > **(4)**
% QOL score ≤ 65	30 (19.9%)	1 (2.2%)	0 (0.0%)	1 (6.7%)	28 (43.1%)	**0.000**
Marital Status						0.519
Married	118 (78.1%)	35 (76.1%)	19 (76.0%)	14 (93.3%)	50 (76.9%)	
Others	33 (21.9%)	11 (23.9%)	6 (24.0%)	1 (6.7%)	15 (23.1%)	
Primary education					χ^2^ = 6.683	0.083
≤6 years	85 (56.3%)	19 (41.3%)	14 (56.0%)	10 (66.7%)	42 (64.6%)	
>6 years	66 (43.7%)	27 (58.7%)	11 (44.0%)	5 (33.3%)	23 (35.4%)	
Employment Status						0.059
Unemployed	132 (87.4%)	38 (82.6%)	19 (76.0%)	15 (100.0%)	60 (92.3%)	
Employed	19 (12.6%)	8 (17.4%)	6 (24.0%)	0 (0.0%)	5 (7.7%)	
Religion						0.564
nil	49 (32.5%)	14 (30.4%)	8 (32.0%)	4 (26.7%)	23 (35.4%)	
Buddhism	65 (43.0%)	23 (50.0%)	10 (40.0%)	6 (40.0%)	26 (40.0%)	
Christianity	3 (2.0%)	0 (0.0%)	1 (4.0%)	1 (6.7%)	1 (1.5%)	
Catholicism	12 (7.9%)	1 (2.2%)	1 (4.0%)	3 (20.0%)	79 (10.8%)	
Taoism	21 (13.9%)	8 (17.4%)	5 (20.0%)	1 (6.7%)	7 (10.8%)	
miscellaneous	1 (0.7%)	0 (0.0%)	0 (0.0%)	0 (0.0%)	1 (1.5%)	
Monthly income (USD)						0.494
<300	87 (57.6%)	24 (52.2%)	12 (48.0%)	10 (66.7%)	41 (63.1%)	
300–1000	37 (24.5%)	9 (19.6%)	7 (28.0%)	4 (26.7%)	17 (26.2%)	
1000–2000	17 (11.3%)	8 (17.4%)	4 (16.0%)	1 (6.7%)	4 (6.2%)	
>2000	10 (6.6%)	5 (10.9%)	2 (8.0%)	0 (0.0%)	3 (4.6%)	
Living						0.335
alone	5 (3.3%)	2 (4.3%)	0 (0.0%)	1 (6.7%)	2 (3.1%)	
with family	139 (92.1%)	44 (95.7%)	22 (88.0%)	13 (86.7%)	60 (92.3%)	
other	7 (4.6%)	0 (0.0%)	3 (12.0%)	1 (6.7%)	3 (4.6%)	
Diabetes mellitus	84 (55.6%)	27 (58.7%)	14 (56.0%)	8 (53.3%)	35 (53.8%)	0.961
BUN (mg/dL)	75.2 ± 20.9	79.7 ± 19.2	78.8 ± 21.8	75.1 ± 22.6	70.8 ± 21.1	0.129
Creatinine (mg/dL)	10.34 ± 2.58	11.3 ± 2.5	10.7 ± 2.7	9.9 ± 2.0	9.7 ± 2.5	**0.008** **(1)** > **(4)**
Sodium (mmol/L)	136.9 ± 4.1	137.3 ± 2.6	137.0 ± 4.2	137.0 ± 4.6	136.5 ± 4.8	0.806
Potassium (mmol/L)	4.44 ± 0.97	4.5 ± 0.8	4.6 ± 0.8	4.5 ± 1.0	4.3 ± 1.1	0.660
Calcium (mg/dL)	9.30 ± 0.87	9.3 ± 0.9	9.3 ± 0.7	9.0 ± 1.1	9.4 ± 0.9	0.610
Phosphate (mg/dL)	4.90 ± 1.47	4.9 ± 1.4	5.5 ± 1.5	5.5 ± 1.4	4.5 ± 1.4	**0.015**
Ca x P product	45.70 ± 14.65	46.1 ± 13.9	50.8 ± 16.3	49.1 ± 11.8	42.7 ± 14.7	0.081
Albumin (g/dL)	3.65 ± 0.37	3.8 ± 0.3	3.7 ± 0.4	3.6 ± 0.3	3.5 ± 0.4	**0.001** **(1)** > **(4)**
Glucose (mg/dL)	153.2 ± 65.8	153.3 ± 57.6	147.4 ± 69.9	157.1 ± 49.8	154.5 ± 73.5	0.966
GOT (IU/L)	18.4 ± 10.1	18.3 ± 9.4	18.0 ± 6.0	20.9 ± 20.3	18.2 ± 8.5	0.802
GPT (IU/L)	19.1 ± 20.0	18.2 ± 15.5	18.2 ± 10.8	25.1 ± 47.3	18.8 ± 15.1	0.689
Kt/V of urea	1.34 ± 0.22	1.4 ± 0.3	1.4 ± 0.2	1.3 ± 0.1	1.3 ± 0.2	0.160
Dialysis vintage (years)	4.5 ± 3.9	3.9 ± 3.8	4.8 ± 3.2	5.6 ± 4.4	4.7 ± 4.1	0.437
ALKP (U/L)	307.6 ± 138.6	297.9 ± 160.8	315.5 ± 137.7	333.1 ± 116.7	305.6 ± 128.4	0.846
Triglyceride (mg/dL)	169.8 ± 111.9	164.8 ± 81.5	158.7 ± 128.5	175.1 ± 94.7	176.3 ± 128.1	0.901
Cholesterol (mg/dL)	159.8 ± 34.9	166.6 ± 39.4	153.3 ± 35.1	150.7 ± 24.2	159.6 ± 33.2	0.306
Ferritin (ng/mL)	460.8 ± 444.5	410.9 ± 215.1	644.4 ± 920.4	391.8 ± 437.9	441.4 ± 233.5	0.149
i-parathyroid hormone (pg/mL)	338.0 ± 271.4	338.9 ± 272.2	453.7 ± 346.5	278.3 ± 201.7	306.7 ± 244.0	0.104
Hemoglobin (g/dL)	10.78 ± 1.51	10.9 ± 1.5	10.6 ± 1.4	10.8 ± 1.8	10.8 ± 1.5	0.950
Platelet (k/µL)	177.1 ± 64.9	170.7 ± 45.9	169.5 ± 64.9	208.4 ± 80.1	177.3 ± 71.6	0.234

Data are presented as the mean ± standard deviation, or as the count number (percentage) in each TDQ subgroup. The p values are from Chi square test, Fisher’s exact test, or ANOVA among the 4 subgroups with post hoc comparisons performed using the Scheffe test (shown if significant, p < 0.05). Between-group comparisons of the 3-year mortality are performed by z-tests, and those with p < 0.05 are shown. The abbreviations are listed in Table 1.

### Affective and cognitive symptoms of depression are better predictors of long-term mortality than somatic symptoms in patients on chronic hemodialysis

**Figure 1:**
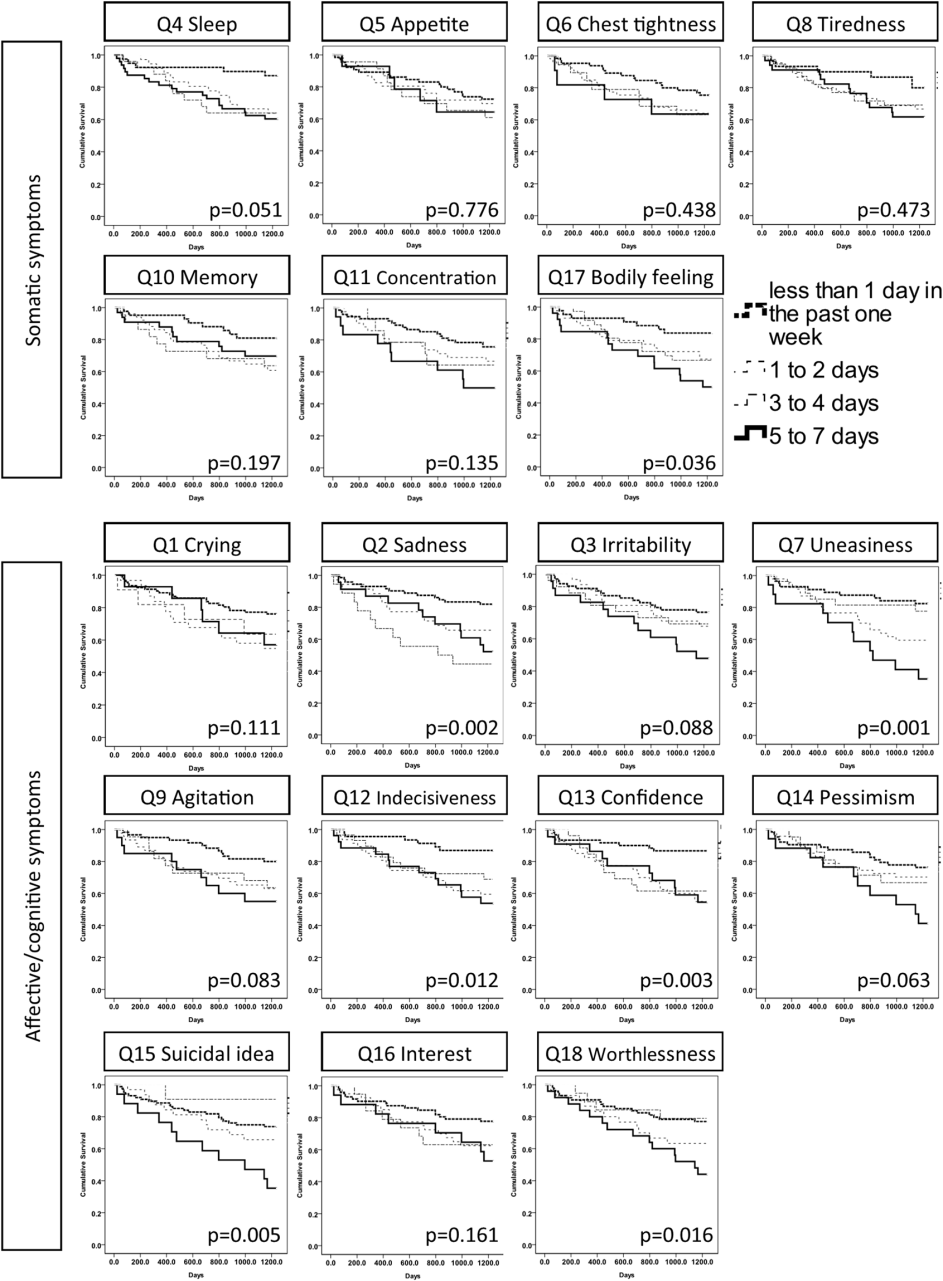
Kaplan−Meier survival curves by individual depressive symptoms. The subjects are divided into 4 groups according to the frequency of the symptoms (the answers to each question). The individual p values of the log-rank test are shown.

**Figure 2:**
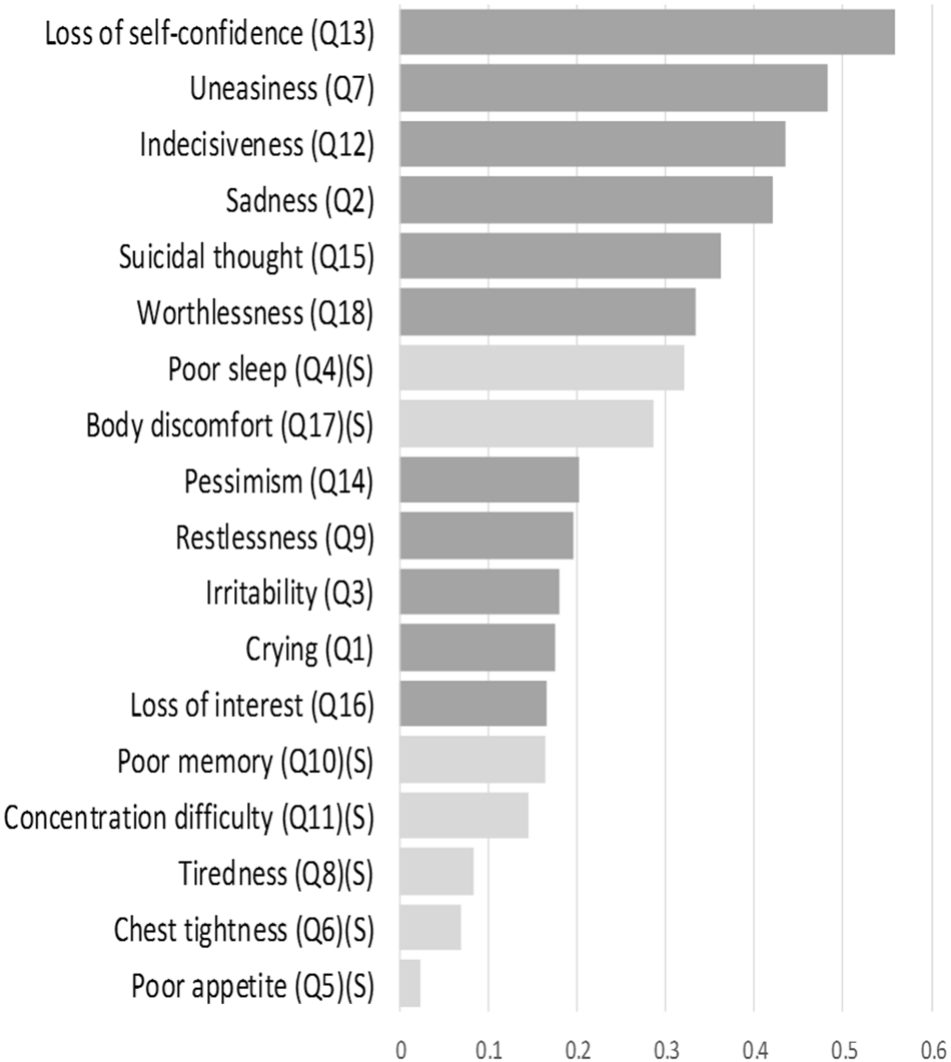
Information value plot of each question in the Taiwanese Depression Questionnaire in predicting mortality. Questions regarding somatic symptoms are indicated by S in parentheses (S) and lighter shades in the bars.

Only question 17 in the somatic subscale (bodily discomfort) showed a significant odds ratio for mortality. Conversely, many questions in the affective and cognitive subscale (i.e., 2, 7, 12, 13 and 14) were associated with mortality. Those questions with significant hazard ratios of mortality were the same ones with significant odds ratios (Table 3). Kaplan-Meier survival analysis identified questions 17 (somatic), 2, 7, 12, 13 15 and 18 (affective/cognitive) as associated with mortality (Table 3 and Fig. 1). The top four questions with the highest information values in prediction to mortality were questions 13, 7, 12 and 2 (Fig. 2). These questions were also the affective and cognitive questions that showed statistical significance in all three of the aforementioned analyses (Table 3).

**Table 3:** Statistical analyses of the association between patient mortality and individual items in the Taiwanese Depression Questionnaire.

		Logistic regression, Non-adjusted			Logistic regression, adjusted by age, albumin			Cox regression, adjusted by age, albumin			Log rank test in Kaplan-Meier analysis
		Exp(B)	95% C.I		Exp(B)	95% C.I		HR	95% C.I		
Somatic symptoms	4	**1.458**	**1.077**	**1.975**	1.323	0.957	1.830	1.259	0.965	1.641	0.051
	5	1.161	0.819	1.644	1.011	0.693	1.473	1.029	0.764	1.386	0.776
	6	1.275	0.878	1.852	1.183	0.792	1.766	1.167	0.852	1.599	0.438
	8	1.231	0.883	1.718	1.014	0.699	1.471	1.037	0.772	1.392	0.473
	10	1.145	0.839	1.561	1.000	0.716	1.397	1.049	0.808	1.361	0.197
	11	**1.423**	**1.023**	**1.980**	1.299	0.918	1.839	1.284	0.993	1.661	0.135
	17	**1.597**	**1.143**	**2.232**	**1.443**	**1.010**	**2.061**	**1.351**	**1.022**	**1.785**	**0.036**
Affective and cognitive symptoms	1	1.315	0.943	1.834	1.231	0.865	1.752	1.147	0.876	1.503	0.111
	2	**1.640**	**1.204**	**2.233**	**1.535**	**1.101**	**2.140**	**1.373**	**1.074**	**1.754**	**0.002**
	3	**1.420**	**1.046**	**1.929**	1.314	0.951	1.815	1.232	0.958	1.583	0.088
	7	**1.632**	**1.157**	**2.301**	**1.489**	**1.029**	**2.156**	**1.380**	**1.031**	**1.849**	**0.001**
	9	**1.421**	**1.026**	**1.968**	1.347	0.954	1.901	1.273	0.979	1.657	0.083
	12	**1.549**	**1.114**	**2.156**	**1.417**	**1.001**	**2.006**	**1.318**	**1.009**	**1.723**	**0.012**
	13	**1.638**	**1.187**	**2.262**	**1.519**	**1.078**	**2.140**	**1.333**	**1.030**	**1.725**	**0.003**
	14	**1.513**	**1.080**	**2.120**	**1.509**	**1.045**	**2.178**	**1.365**	**1.039**	**1.794**	0.063
	15	**1.429**	**1.033**	**1.977**	1.353	0.949	1.928	1.239	0.946	1.625	**0.005**
	16	**1.421**	**1.024**	**1.973**	1.357	0.954	1.931	1.242	0.954	1.616	0.161
	18	**1.475**	**1.091**	**1.995**	1.358	0.986	1.870	1.242	0.972	1.586	**0.016**

The odds ratios are calculated by the beta exponential [Exp(B)]. P values for the log-rank test of Kaplan-Meier survival analysis are presented. The question order is rearranged into the somatic and affective/cognitive subgroups. C.I. indicates the confidence interval.

### Affective and cognitive symptoms of depression are more closely associated with lower quality of life than somatic symptoms of depression

When ranked according to the Spearman’s rank order correlation coefficient (rho), affective/cognitive symptoms were more significantly correlated with lower quality of life, as compared to somatic symptoms (p < 0.05, Mann-Whitney U test; Table 4). In other words, the questions with higher correlation coefficients were primarily regarding affective/cognitive symptoms.

**Table 4:** Spearman’s rank order correlation coefficient (rho) between each question in the Taiwanese Depression Questionnaire and the total score of the WHO Quality of Life Questionnaire-brief version.

Item number	Affective/cognitive or Somatic (U = 10, p < 0.05)	Item description	Spearman correlation coefficient (rho)
13	A	Loss of self-confidence	−0.703
2	A	Sadness	−0.669
18	A	Worthlessness	−0.668
7	A	Uneasiness	−0.643
3	A	Irritability	−0.638
16	A	Loss of interest	−0.629
15	A	Suicidal thought	−0.609
12	A	Indecisiveness	−0.608
17	S	Bodily discomfort	−0.605
8	S	Tiredness	−0.581
9	A	Restlessness	−0.578
4	S	Poor sleep	−0.550
1	A	Crying	−0.549
6	S	Chest tightness	−0.540
10	S	Poor memory	−0.527
14	A	Pessimism	−0.522
11	S	Concentration difficulty	−0.504
5	S	Poor appetite	−0.417

All p values are less than 0.001. The items are listed in order from the highest negative correlation. The Mann-Whitney U test is used to compare the order between the affective/cognitive items and the somatic items.

### Affective and cognitive symptoms of depression are associated with distinct demographic and biochemical characteristics as compared to somatic symptoms of depression

When comparing somatic and affective/cognitive subscale scores based on different demographic characteristics (Table 5), women appeared to have higher somatic scores (p = 0.011) and affective/cognitive scores (p = 0.051) than men. Less education was also associated with higher somatic and affective/cognitive scores. A lower income appeared to be a risk factor for a high somatic subscale score (p = 0.019) but not for the affective/cognitive subscale score (p = 0.179). When correlating subscale scores and the biochemical parameters (Table 6), we found that the somatic subscale score was significantly correlated with low creatinine, albumin and blood urea nitrogen (BUN) levels, as well as with age and the dialysis vintage. The affective/cognitive subscale score was significantly correlated with low creatinine, albumin, BUN and phosphate levels but not with age (p = 0.248) or the dialysis vintage (p = 0.097). According to multiple linear regression to identify the important independent association factors (Table 7), somatic symptoms were associated with low serum creatinine and albumin levels and the dialysis vintage, whereas affective/cognitive symptoms were associated with a low albumin level.

**Table 5:** Scores of the somatic subscale and the affective/cognitive subscale of the Taiwanese Depression Questionnaire based on demographic characteristics.

	Somatic subscale	*p* value of *t*-test, or ANOVA and post hoc Scheffe test	Affective/Cognitive subscale	*p* value of *t*-test, or ANOVA and post hoc Scheffe test
Gender		**0.011**		0.051
Men (74)	7.15 ± 5.20		9.01 ± 8.52	
Women (77)	9.42 ± 5.67		12.04 ± 10.34	
Marital status		0.685		0.210
Married (118)	8.19 ± 5.28		9.97 ± 9.04	
Other (33)	8.70 ± 6.48		12.67 ± 11.2	
Education		**0.021**		**0.044**
≤6 years (85)	9.24 ± 5.18		11.92 ± 9.9	
>6 years (66)	7.11 ± 5.80		8.8 ± 8.93	
Occupation		0.352		0.269
Unemployed (132)	8.48 ± 5.44		10.92 ± 9.45	
Employed (19)	7.05 ± 6.23		8.05 ± 10.41	
Religion		F = 0.284, p = 0.921		F = 0.618, p = 0.686
nil (49)	8.53 ± 5.15		11.16 ± 9.62	
Buddhism (65)	7.85 ± 6.08		10.28 ± 9.67	
Christianity (3)	9.67 ± 3.06		9.33 ± 8.08	
Catholicism (12)	9.67 ± 4.23		14 ± 10.05	
Taoism (21)	8.19 ± 5.99		8.19 ± 9.52	
Miscellaneous (1)	9.00		11	
Monthly income (USD)		F = 3.491, **p** = **0.019**		F = 1.657, p = 0.179
<300 (17)	8.43 ± 5.16	(2) > (3)	10.8 ± 9.29	
300–1000 (10)	10.00 ± 6.33		12.43 ± 10.84	
1000–2000 (87)	5.47 ± 5.19		6.76 ± 7.77	
>2000 (37)	5.80 ± 4.05		7.9 ± 8.88	
Living		F = 0.043, p = 0.958		F = 0.749, p = 0.475
Alone (5)	9.00 ± 5.79		11.00 ± 10.15	
with family (139)	8.27 ± 5.62		10.32 ± 9.51	
Other (7)	8.43 ± 4.35		14.86 ± 11.16	
Diabetes mellitus		0.314		0.286
No (67)	8.82 ± 5.85		11.51 ± 10.41	
Yes (84)	7.89 ± 5.29		9.8 ± 8.86	

Data are presented as the mean ± standard deviation. USD: US dollars.

**Table 6:** Pearson’s correlation coefficient and p values of the biochemical data with the somatic subscale and the affective/cognitive subscale.

	Pearson correlation coefficient with Somatic subscale	*p* value	Pearson correlation coefficient with Affective/Cognitive subscale	*p* value
Age	0.193	**0.018**	0.095	0.248
BUN	−0.155	**0.057**	−0.221	**0.006**
Creatinine	−0.347	**0.000**	−0.308	**0.000**
Sodium	−0.076	0.354	−0.080	0.330
Potassium	−0.117	0.152	−0.142	**0.083**
Calcium	0.040	0.629	0.049	0.552
Phosphate	−0.106	0.195	−0.179	**0.028**
Ca x P product	−0.080	0.327	−0.142	**0.082**
Albumin	−0.317	**0.000**	−0.298	**0.000**
Glucose	0.002	0.981	0.004	0.960
GOT	0.111	0.176	0.020	0.810
GPT	0.107	0.190	0.023	0.783
Kt/V of urea	−0.102	0.213	−0.108	0.187
Dialysis vintage (years)	0.154	**0.058**	0.136	**0.097**
ALKP	0.113	0.169	0.034	0.676
Triglyceride	−0.002	0.984	0.008	0.922
Cholesterol	−0.111	0.176	−0.083	0.312
Ferritin	0.040	0.627	−0.065	0.429
i-parathyroid hormone	0.034	0.676	−0.077	0.347
Hemoglobin	−0.029	0.725	0.051	0.531
Platelet	0.023	0.783	0.057	0.486

p values lower than 0.1 are labeled in bold. ALKP: alkaline phosphatase, BUN: blood urea nitrogen, Ca x P: calcium and phosphate product, GOT: glutamic oxaloacetic transaminase, GPT: glutamic pyruvic transaminase, i-parathyroid hormone: intact parathyroid hormone, Kt/V: dialysis adequacy by dialyzer clearance of urea (K) times time (t) divided by volume (V) of the urea distribution.

**Table 7:** Multiple linear regression model of select variables with the somatic or affective/cognitive subscales.

	Multiple linear regression model with Somatic subscale				Multiple linear regression model with Affective/cognitive subscale			
	Standardized beta	95% confidence interval		*p* value	Standardized beta	95% confidence interval		*p* value
Creatinine	−0.229	−0.860	−0.123	**0.009**	−0.175	−1.315	0.017	0.056
Albumin	−0.203	−5.608	−0.493	**0.020**	−0.205	−9.780	−0.897	**0.019**
Dialysis vintage	0.157	0.011	0.435	**0.039**	0.137	−0.039	0.711	0.079
Age	0.058	−0.040	0.087	0.469	ni	ni	ni	ni
Potassium	ni	ni	ni	ni	−0.060	−2.157	0.964	0.451
Phosphate	ni	ni	ni	ni	−0.068	−1.523	0.641	0.422

ni: not included.

## Discussion

There are 4 major findings in this article. First, in dialysis patients, depressive symptoms are associated with mortality, even in patients who have elevated, but subthreshold scores on the TDQ.

Second, affective and cognitive symptoms, including sad mood (Q2), tenseness (Q7), indecisiveness (Q12), and low self-confidence (Q13), rather than somatic symptoms, predicted long-term mortality rates. Third, affective and cognitive symptoms were more correlated with quality of life than somatic symptoms. Fourth, distinct demographic and biochemical characteristics are associated with affective and cognitive symptoms of depression as compared to somatic symptoms of depression. The association factors for the affective and cognitive symptoms included lower education and serum albumin levels, while the somatic symptoms were associated with female gender, lower education level, lower income, low serum creatinine and albumin levels and dialysis vintage.

The finding that subthreshold depressive symptoms are also associated with higher mortality rate is important and has not previously reported, to our knowledge. All previous studies that have demonstrated the link between depression and mortality have examined the outcomes between depressed and non-depressed subjects. In contrast, we divided our subjects into 4 groups based on their depression scores: normal (0–8), low abnormal (9–14), high abnormal (15–18), and depressive (19–54). Individuals with high abnormal scores showed a mortality rate that was as high as the rate for those regarded as depressive. This finding implies the importance of early intervention in those with high abnormal scores.

Only two previous studies have demonstrated an association between the cognitive symptoms in depression and mortality in hemodialysis patients. Kimmel *et al*.^25^ found that the relative risk of death in chronic hemodialysis patients was 1.32 (95% confidence interval 1.13 to 1.55) based on the Beck Depression Inventory scores and 1.23 (95% confidence interval 1.05 to 1.43) based on the Cognitive Depression Index. Another study by Peterson *et al*.^26^ found that the CDI scores at one-year follow-up were already different between the surviving and non-surviving patients on hemodialysis or peritoneal dialysis, although their BDI scores did not differ. These two studies highlight the importance of cognitive symptoms in association with mortality in dialysis patients; however, their results should not be interpreted as indicating that the somatic symptoms are less significant.

The reason why the somatic indicators fail to predict mortality in hemodialysis patients is unknown. One possibility is that hemodialysis treats uremia-related somatic symptoms, such as fatigue, poor appetite and concentration difficulty, and might therefore minimize the impact of somatic symptoms in patients on hemodialysis. Our analysis of the association factors supports this notion. The somatic subscale score is not correlated with two surrogate uremic markers, blood urea nitrogen and creatinine. Consequently, the somatic symptoms may become indistinguishable between the survivors and non-survivors. In contrast, in patients with myocardial infarction or chronic heart failure^24,29^, somatic/affective symptoms rather than cognitive/affective symptoms are associated with mortality and cardiovascular outcomes. Somatic symptoms such as fatigue and appetite may directly reflect the physical condition as well as the disease severity of these patients, and therefore, the somatic symptoms are associated with mortality.

Another hypothesis is that perception of illness may be correlated with mortality, a dimension which is potentially captured through affective/cognitive symptoms rather than somatic symptoms. In other words, patients with more severe disease may also have more prominent cognitive symptoms, and therefore, cognitive symptoms are presumably more associated with mortality. Sacks^30^ reported that the correlation coefficient between the Cognitive Depression Index and the perception of illness score was 0.49 (p < 0.001) in dialysis patients, whereas the correlation coefficient was 0.67 (p < 0.001) between the BDI and the perception of illness score. However, no data were presented regarding the association between the somatic symptoms and the perception of illness.

**Figure 3:**
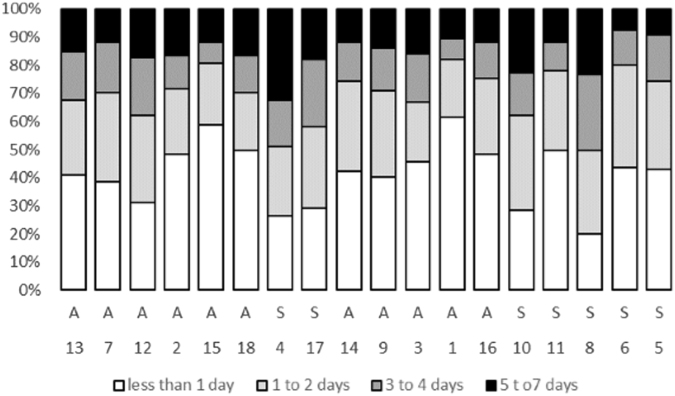
Bar chart of the proportion of the answers to each question of the Taiwan Depression Questionnaire. The order of the questions is based on the information values with the highest on the left. A stands for affective/cognitive items; S for somatic items. The answers are listed as the number of days that the symptoms are present in the past week.

Another possible argument is that self-report scales have lower specificity for detecting only patients with clear depressive disorders, and therefore, the study sample might overestimate the prevalence of depressive symptoms by including patients with only somatic symptoms related to their hemodialysis. That is, the somatic symptoms of depression may be found in so many patients and with such a high degree of severity that these symptoms cannot differentiate between survivors and non-survivors. However, our analysis of the frequency distribution of the responses to each question counters this argument (Fig. 3). In fact, for most questions, far more participants did not report symptoms than reported having symptoms. The frequency distribution patterns for each question were also not significantly related to the associations of the questions with mortality.

We used 4 different analytic tools to identify the questions that might predict mortality (Table 3, Figs 1 and 2). The tools differ mathematically; therefore, the questions that pass all tests should have high predictive power. Interestingly, the logistic regression analyses and Cox regression models identified exactly the same 5 affective and cognitive questions that were associated with mortality. The Kaplan-Meier analysis illustrated the survival difference from the responses to each question, and the questions that displayed statistical significance were very similar to those found by the aforementioned 2 methods. There were 4 affective and cognitive questions showing statistical significance in all three methods, and they were the same ones that topped the information value plot. Therefore, we are confident in reporting the importance of four key affective and cognitive symptoms, which are sad mood (Q2), tenseness (Q7), indecisiveness (Q12), and low self-confidence (Q13), to predict mortality.

Our results are the first to demonstrate that quality of life is more correlated with affective and cognitive symptoms than with somatic symptoms. The top three items that showed high correlation coefficients were loss of self-confidence, sadness, and worthlessness, all of which were negative thoughts in terms of depression symptomatology. Somatic symptoms displayed lower correlations. This result emphasizes how easily quality of life can be influenced by a melancholic mood. Another interesting point was that the question regarding body pain was the only somatic symptom that was associated with mortality, and was also the most influential question relating somatic symptoms to quality of life.

Despite the challenges in correctly diagnosing depression in end-stage renal disease patients due to overlapping symptomatology, our findings appear to support the existence of depression as a distinct and clinically significant entity in this population. The finding that the somatic scores are associated with many extrinsic factors implies that the somatic symptoms of depression may be secondary in hemodialysis patients. However, the data showing lack of associations of the affective and cognitive scores with various factors are compatible with the fact that depression is currently regarded as more of a neurobiological disease rather than an illness only caused by psychological or environmental stresses^31^. Therefore, depression in hemodialysis patients should not be overshadowed by their somatic complaints.

This study has several limitations. First, the symptoms of depression were defined using a self-reported questionnaire instead of diagnosis by a psychiatrist. We tried to minimize individual differences in interpreting the questions by acquiring the answers through interviews performed by registered nurses, instead of simply giving the questionnaire to our subjects. The second concern is the low sample number. Although a larger sample size might have demonstrated significant associations with somatic symptoms of depression, this association would be likely less significant than that of affective and cognitive symptoms with mortality. Nevertheless, a larger sample size might allow more in-depth subgroup analyses. For example, we need to recruit more subjects to determine whether the somatic symptoms are associated with mortality in individuals with a shorter dialysis vintage. Third, the Likert scale, which ranges from 0 to 3, is based on the frequency of a certain symptom over the past week, and therefore, the severity is not measured by degree. A symptom that is severe but manifests only once a week is by definition scored only one point, which makes these symptoms less useful in predicting the outcomes. Fourth, the study did not adjust for anxiety. It is known that anxiety is associated with an increased mortality rate in patients undergoing hemodialysis^32^. DSM-5 (the Diagnostic and Statistical Manual of Mental Disorders, 5th Edition) criteria for generalized anxiety disorder include “increased muscle aches or soreness,” which are overlapped with the physical complaints represented by questions 6 (chest tightness) and 17 (headache, dizziness, palpitation and abdominal distress) in the somatic subscale of the TDQ. The association we found in this study between question 17 and mortality might actually reflect the association between anxiety and mortality.

In conclusion, we found that affective and cognitive symptoms rather than somatic symptoms of depression were associated with long-term mortality and quality of life in patients undergoing chronic hemodialysis. As such, systematic assessment of affective and cognitive symptoms of depression should be undertaken in patients on chronic hemodialysis. Patients with depressive symptoms, as well as those with elevated subthreshold symptoms, should be referred for timely and appropriate intervention.

## Methods

In this prospective cohort study, we followed the patients for more than three years to assess mortality. The ethics committee of the Institutional Research Board at the National Taiwan University Hospital Hsin-Chu Branch approved the proposal before the study. All methods were carried out in accordance with relevant guidelines and regulations. Informed consent was obtained from all participants. They were recruited from a hemodialysis facility at a secondary hospital from September 2012 to September 2013. This 819-bed hospital is government-run and located in an urban area of northern Taiwan. The inclusion criteria were (1) having undergoing at least 6 months of hemodialysis treatment, (2) being older than or equal to 20 years of age, and (3) being oriented to person and place and capable of answering the questionnaires. We excluded patients with advanced cancer (stage 4) and those with a tracheostomy. A total of 193 patients were eligible, from whom a random sample of 160 patients was chosen. Several patients declined to participate in the study due to privacy concerns. Once the informed consent was obtained, the interview was initiating by asking the patients to report their name, date of birth, age, gender, residential area (county, city and district) and 10-digit identification number, information that was corroborated with the chart by the interviewer. If these basic orientation questions were answered incorrectly, they were excluded from the study. Overall, 151 subjects signed the informed consent forms and completed the interview.

We used the Taiwanese Depression Questionnaire to assess depression by asking subjects the frequency with which they had experienced 18 conditions within the past week. The frequency scale is as follows: 0 (never or seldom; fewer than one day per week), 1 (sometimes; 1–2 days per week), 2 (often; 3–4 days per week) and 3 (very often or always; 5–7 days per week). A total score equal to or higher than 19 had been validated to indicate depression, with 89% sensitivity and 92% specificity^28^. This questionnaire adopted a few culturally relevant questions, such as chest tightness (question 6) and bodily discomfort such as headache, dizziness, palpitation, or abdominal distress (question 17)^33^, as depressed Taiwanese patients had been shown to demonstrate predominant somatic manifestations of illness. The TDQ and the BDI were compared for the validity in evaluating depression in patients with chronic pain^34^. Items from both questionnaires are listed for comparison in Table S1 (the English version of the questions were given by the original authors of the TDQ^34^). Questions regarding “past failure,” “guilty feelings,” “punishment feelings,” and “loss of interest in sex” in the BDI were replaced. Question 10, poor memory or forgetfulness, was classified as a somatic item according to Freudenreich *et al*.^19^, although it was regarded as a cognitive item in other literatures^35^. Question 7, “I felt uneasy, uncomfortable,” was classified as a affective/cognitive item as it asked for being “not in peaceful mind,” or having “psychological stress” in Chinese language.

Quality of life was assessed by the World Health Organization Quality of Life assessment questionnaire-brief version (WHOQOL-BREF). This questionnaire is composed of 28 questions that cover four major functions, or domains: physical health, psychological health, social relationships and environment. A higher score indicates a better quality of life. Three registered nurses interviewed the subjects face-to-face to complete the questionnaires. Patient demographic, biochemistry and hemodialysis-related data were also collected.

We used SPSS 20 (IBM, Armonk, New York, United States) to perform the statistical analyses except for the calculation of importance values. The statistician did not participate in data collection, and had no knowledge about which individuals were dead or alive in the final de-identified version of the data. Categorical data were tested using Chi-square test; or Fisher’s Exact test if the cell number was less than five. Proportions between two groups were compared with z-tests. Analysis of variance (ANOVA) was used to compare biochemical data among three or more groups, and the Scheffe method was used for the post hoc analysis. We calculated the odds ratio of mortality according to the answers to each question in the Taiwanese Depression Questionnaire using logistic regression analysis with or without adjustments for age and albumin, which are two major determinants of mortality. The hazard ratios of mortality according to the answers to each question in the TDQ were obtained by Cox proportional regression modeling with adjustments for age and albumin. Log-rang test using Kaplan-Meier survival analysis was used to examine whether those who answered differently (4 different answers to each question) also have different survival rates. Weights of evidence (WOEs) were calculated using the formula WOE = natural logarithm (% of survival/% of death). The information value was the sum of (% of survival/% of death) times WOE. The Mann-Whitney U test was used to compare the order between the affective/cognitive symptoms and the somatic symptoms listed in order by the Spearman rho values. Multiple regression analysis was used to identify independent association factors. Spearman’s rank order correlation coefficient, named rho, was calculated to test the correlation between the answers of each TDQ item and the quality of life score. *p* value less than 0.05 was considered statistically significant.

## Electronic supplementary material

Supplementary Table
