# Supplementary material for: Affective and cognitive rather than somatic symptoms of depression predict 3-year mortality in patients on chronic hemodialysis
Source: Sci Rep. 2018 Apr 12;8:5868. doi: 10.1038/s41598-018-24267-5 (PMC5897563; doi:10.1038/s41598-018-24267-5)
Supplement: Supplementary file 1 — Supplementary Table [file 41598_2018_24267_MOESM1_ESM.pdf]

Affective and cognitive rather than somatic symptoms of depression predict 3-year mortality in patients on chronic hemodialysis

Hui-Teng Cheng, Miao-Chun Ho, Kuan-Yu Hung

Table S1. Questions from the Taiwan Depression Questionnaire and the corresponding questions in the Beck Depression Inventory. The parentheses in the Beck Depression Inventory indicate similar but not identical items. The blanks indicate no corresponding items.

Somatic or affective/ cognitive (A/C)		Taiwan Depression Questionnaire<br>How often have you had the following feeling in the past week?<br>0: never; 1: 1-2 days;<br>2: 3-4 days; 3: 5-7 days	Beck Depression Inventory by item description
A/C	1	I often felt like crying	10. Crying
A/C	2	I felt sad and depressed	1. Sadness
A/C	3	I felt more irritable/agitated (prone to get angry) than I used to	17. Irritability<br>11. Agitation
Somatic	4	I had trouble sleeping	16. Changes in sleep pattern
Somatic	5	I did not want to eat<br>(I had a poor appetite)	18. Changes in appetite
Somatic	6	I had chest tightness	
A/C	7	I felt uneasy, uncomfortable	
Somatic	8	I feel tired and had less energy	15. Loss of energy<br>20. Tiredness or fatigue
A/C	9	I felt upset and restless	(11. Agitation)
Somatic	10	I felt that I had poor memory	
Somatic	11	I could not concentrate when performing tasks	19. Concentration Difficulty
A/C	12	I was slower when thinking of or doing things than I used to be	13. Indecisiveness
A/C	13	I felt less confident than I used to	(7. Self-dislike)<br>(8. Self-criticalness)
A/C	14	I tended to think of the negative side	2. Pessimism
A/C	15	I felt miserable and even wanted to die	9. Suicidal thoughts or wishes
A/C	16	I lost interest in everything	4. Loss of pleasure<br>12. Loss of interest
Somatic	17	I felt sick in part of my body (headache, dizziness, palpitation, or abdominal distress … etc.)	
A/C	18	I felt worthless	14. Worthlessness
			2. Past failure
			5. Guilty feelings
			6. Punishment feelings
			21. Loss of interest in sex
